# Sensitivity of the Norwegian and Barents Sea Atlantis end-to-end ecosystem model to parameter perturbations of key species

**DOI:** 10.1371/journal.pone.0210419

**Published:** 2019-02-08

**Authors:** Cecilie Hansen, Kenneth F. Drinkwater, Anne Jähkel, Elizabeth A. Fulton, Rebecca Gorton, Mette Skern-Mauritzen

**Affiliations:** 1 Institute of Marine Research, Bergen, Norway; 2 CSIRO Oceans and Atmosphere, Hobart, Tasmania, Australia; 3 Centre for Marine Socioecology, University of Tasmania, Hobart, Tasmania, Australia; Aristotle University of Thessaloniki, GREECE

## Abstract

Using end-to-end models for ecosystem-based management requires knowledge of the structure, uncertainty and sensitivity of the model. The Norwegian and Barents Seas (NoBa) Atlantis model was implemented for use in ‘what if’ scenarios, combining fisheries management strategies with the influences of climate change and climate variability. Before being used for this purpose, we wanted to evaluate and identify sensitive parameters and whether the species position in the foodweb influenced their sensitivity to parameter perturbation. Perturbing recruitment, mortality, prey consumption and growth by +/- 25% for nine biomass-dominating key species in the Barents Sea, while keeping the physical climate constant, proved the growth rate to be the most sensitive parameter in the model. Their trophic position in the ecosystem (lower trophic level, mid trophic level, top predators) influenced their responses to the perturbations. Top-predators, being generalists, responded mostly to perturbations on their individual life-history parameters. Mid-level species were the most vulnerable to perturbations, not only to their own individual life-history parameters, but also to perturbations on other trophic levels (higher or lower). Perturbations on the lower trophic levels had by far the strongest impact on the system, resulting in biomass changes for nearly all components in the system. Combined perturbations often resulted in non-additive model responses, including both dampened effects and increased impact of combined perturbations. Identifying sensitive parameters and species in end-to-end models will not only provide insights about the structure and functioning of the ecosystem in the model, but also highlight areas where more information and research would be useful—both for model parameterization, but also for constraining or quantifying model uncertainty.

## Introduction

Comprehensive end-to-end ecosystem models are key tools for implementation of an ecosystem-based approach to management (e.g. [1,2,3,4]). These models are typically developed to assess our ecosystem knowledge, to study ecosystem structure and functioning, and to test ecosystem responses to human impact ([5,6]. Likewise, the ability to perform ‘what-if’ scenarios makes ecosystem models powerful tools to determine feasible, effective and efficient options for cross-sector ecosystem management, as well as in the field of ecosystem-based fisheries management[7,8].

Atlantis is one such comprehensive end-to-end ecosystem model, including the natural system from physics to fish, whales and seabirds, fishing and other human activities impacting the ecosystem[9]. Recently, an Atlantis ecosystem model was parameterized for the Nordic and Barents Seas (NoBa;[10]). While the model is expected to become a significant tool for simulating human impacts and testing management strategies, a core area of use will be to investigate how the two most important drivers in these high latitude ecosystems, climate and fisheries, interact and impact management strategies. This model, covering an area of 4 million km^2^, is spatially resolved and composed of 52 species and functional groups (hereafter referred to as components). The processes affecting e.g. nutrient recycling, physiology, population dynamics, distributions and species interactions are defined through a set of >3000 parameters. These parameters are defined based on estimates from the local ecosystems or other, comparable systems, on general ecological principles, or by tuning ([10, 11]).

The Atlantis model was rated as the best ‘what if’ scenario model by [5]. However, for there to be confidence in its specific performance for an individual system, an assessment of model performance is required[6]. Such assessments can highlight the strengths and deficiencies of an individual model implementation, detect conceptual or coding errors, and assess uncertainties in model results to help further model development ([12, 13]. Sensitivity analyses, aiming to quantify the relationships between model inputs (especially parameters) and the response, are common diagnostic tools used in model development and validation. Sensitivity analyses are used to identify critical and non-critical model parameters. Such analyses help focus efforts around estimation and calibration of the important parameters and provide an assessment of whether the model sensitivity reflects processes that have an impact on the dynamics of the natural ecosystems. Sensitivity analyses can be either *analytical*, by manipulating the model equations directly to establish the input-output relationships, or *empirical*, by measuring the model’s response to perturbations of the inputs [12]. Furthermore, sensitivity analyses can be classified as *local* or *global*, depending on how much of the parameter space is sampled to estimate the response. *Local* methods often explore the model sensitivity to parameter spaces around the most likely point of model operation. *Global* methods explore model sensitivity across a wide range of parameters in the model system. Due to the many feedback mechanisms present in ecosystem models (e.g. through species interactions), model sensitivity to changes in combinations of parameters may reflect both additive and non-additive effects enhancing or dampening model responses to perturbations.

Despite the growing numbers of ecosystem models (> 500 marine ecosystem models are implemented around the world; http://sirs.agrocampus-ouest.fr/EcoBase/, http://atlantis.cmar.csiro.au/), sensitivity analyses are rarely performed, particularly for models that include higher trophic levels. This is due to the long run time of complex ecosystem models, for which the computational cost of sensitivity analyses becomes prohibitively high for many of the global analytical approaches developed to date. In response, smaller searches are more typical—for example, [13] undertook a smaller, local sensitivity analysis in their paper, concluding that the growth rate of large zooplankton was the most sensitive to perturbations.

Here we present a sensitivity analysis of the NoBa Atlantis model. To ensure a manageable run time (the model run time is ~13 hours for a 55-year simulation), we took a *local* and *empirical* approach. We perturbed the parameterization of recruitment, mortality, prey consumption and growth of 9 interacting species and functional groups in the modelled ecosystem. These 9 species and functional groups are recognized by ecologists as key species and groups in the dynamics of these marine ecosystems; 4 groups of zooplankton that convert primary to secondary production; 3 species of pelagic fish that are typical wasp-waist species holding key positions in trophic transfer from lower to higher trophic levels, and the 2 dominating top predators in terms of biomass and consumption; cod and minke whales[14]. Strong interactions have been observed between these groups. The abundance of pelagic fish has varied dramatically over the last four decades, particularly due to repeated collapses in the capelin stock[15]. Zooplankton and pelagic fish abundance are inversely correlated, suggesting a top-down regulation of zooplankton by pelagic fish[16]. However, the strength of this interaction has weakened with the recent warming of the system ([16] and references therein). In this study, however, we are keeping the physics constant, reflecting a cold year (1981). Reduced abundances of pelagic fish have also impacted the top predators of the ecosystem—including recruitment, growth and mortality of cod, marine mammals and seabirds[14]. Hence, we may expect that the model system may be more sensitive to perturbations affecting the pelagic fish than the lower or higher trophic levels. Furthermore, interspecific resource competition may also occur between pelagic fish species[17] and between top predators such as cod and marine mammals[18]. Thus, a sensitivity analysis testing model responses to perturbations of these core ecosystem components is an important means of assessing model sensitivity to parameterization of some of the key players in the dynamics of this ecosystem. As NoBa Atlantis is built particularly for testing climate and fisheries scenarios, confidence in and transparency of the model behaviour with respect to these key components and interactions is of critical importance. This exercise will therefore also help provide the information needed to improve the level of trust of the model by managers who may need its output as a source of information for future planning.

In the sensitivity analysis, the parameters were first perturbed one-by-one, thereafter in combinations, to explore additive and non-additive effects. The aim of this study was to identify sensitive and insensitive parameters, species and functional groups, to i) identify the parameter uncertainties that the model output is most sensitive to, and ii) to explore how model system responses are linked to the food web position of the perturbed species.

## Materials and methods

**Fig 1:**
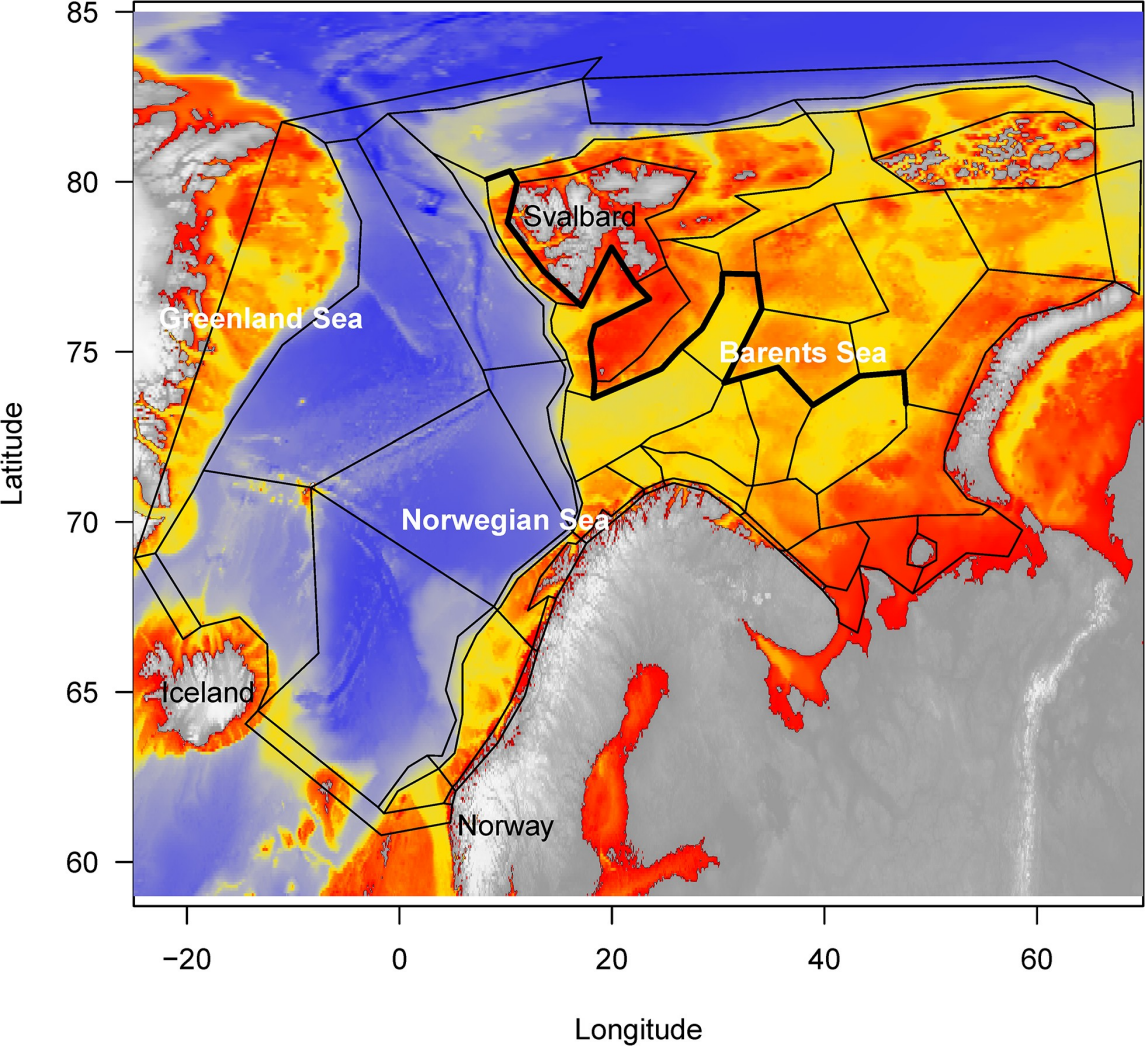
NoBa model domain. The model domain of the Nordic and Barents seas model, including the bathymetry.

The NoBa Atlantis end-to-end model covers the Nordic and Barents Sea (Fig 1), where the Nordic seas include the Greenland, Iceland and Norwegian Sea. The Norwegian and Barents Seas are dominated by contrasting water masses. In the Norwegian Sea, the warm, saline (>35.1) Norwegian Atlantic Slope Current flows northwards along the shelf edge, side-by-side with the fresher Norwegian Coastal Current[19]. Upon reaching the eastern entrance to the Barents Sea, the Norwegian Coastal Current and a branch of the Slope Current enters the Barents Sea. The remainder of the Slope Current heads farther north towards the Arctic. Cold Arctic Water enters the Barents Sea from the north[20]. This Arctic water mass is separated from the Atlantic water by the strong Polar Front (Fig 1). South of the Polar Front, the watermasses represents the warm and saline Atlantic water, whereas the watermasses north of the Polar Front are dominated by Arctic Water. Given the contrasting physical conditions in the two water masses, the ecology of the area is diverse and productive[21], but closely linked through seasonal migrations. Both the Norwegian and Barents seas ecosystems have a limited set of key species, including zooplankton, small pelagic fish, large demersal fish and marine mammals, which dominate in biomass and trophic transfer, and that fluctuate significantly in abundance (e.g. [22,23,24].

### Model description

NoBa Atlantis was set up on a grid with 60 polygons covering the Nordic and Barents Seas (Fig 1). The polygons were defined based on expert knowledge of the topography, hydrographic characteristics and species distributions ([10]). They were designed to have high internal homogeneity in physical and biological properties and high heterogeneity between polygons. Higher spatial resolution was chosen for the Barents Sea to reflect the greater spatial variability in bathymetry of this shelf sea compared to the deeper Norwegian Sea (Fig 1).

Each polygon has up to seven depth levels depending on the mean maximum depth, plus one sediment level. The depth levels are defined as 0–50 m, 50–150 m, 150–250 m, 250–375 m, 375–500 m, 500–1000 m and >1000 m. The radiation (Wm^-2^) and day length is computed within each box based on longitude and latitude and time of year. NoBa Atlantis is run offline with interpolated temperature, salinity and volume fluxes from a Regional Ocean Modelling System (ROMS;[25]. The initial values of nutrients and phytoplankton are derived from the NORWEgian ECological Ocean Modeling system (NORWECOM: [26, 27]).

NoBa Atlantis includes 52 components—49 species/functional groups, representing all trophic levels from bacteria to top predators and 3 detritus groups (Table 1, note scientific names for all the species/groups in the model are listed herein). The components resolved to the species level (N = 26) include the commercially and ecologically important key species (e.g. Atlantic mackerel, Norwegian spring spawning herring and Northeast Atlantic cod) and species that are potentially vulnerable to changes in climate or fisheries (e.g. polar bears and beaked redfish). The components resolved as functional groups (N = 23), represent groups of species (e.g. small pelagics, other demersals) with similar life history, diet and horizontal distribution [10]. The species and groups are connected through a diet matrix, where the maximum potential fraction of prey available for the predator is defined (the realized diet is further determined by spatio-temporal overlap of predator and prey, gape limitation and potentially by habitat dependency and state). The invertebrates are represented as biomass pools, whereas the vertebrates include information about numbers and weights in each polygon at each depth level. All vertebrates are divided into 10 or fewer age classes, depending on the longevity of the species. Atlantis reports weights, numbers and distribution for each of the age classes. Vertebrate weight is divided into structural and reserve weight, where the structural weight represents bones and hard tissue; reserve weight is soft-tissue weight (including fats and gonads). The components change their size depending on availability of food, spawning, preference of growth over spawning, etc. [28]. The reserve weight is coupled to natural mortality and recruitment, e.g. if the components reserve weight drops below a certain limit, spawning may be skipped. Starvation can occur, leading to lower reserve weights and increasing natural mortality.

**Table 1:** Overview over functional groups and species included in NoBa Atlantis.

Full name	Abbreviation	Species included/latin names
Polar Bear	POB	*Ursus maritimus*, Phipps 1774
Killer whale	KWH	*Orcinus orca*, Fitzinger 1860
Sperm whale	SWH	*Physeter macrocephalus*, Linnaeus 1758
Humpback whale	HWH	*Megaptera novaeangliae*, Borowski 1781
Minke whale	MWH	*Balaenoptera acutorostrata*, Lacepede 1804
Fin whale	FWH	*Balaenoptera physalus*, Linnaeus 1758
Bearded seal	BES	*Erignathus barbatus*, Erxleben 1777
Harp seal	HAS	*Pagophilus groenlandicus*, Erxleben 1777
Hooded seal	HOS	*Cystophora cristata*, Erxleben 1777
Ringed seal	RIS	*Phoca hispida*, Schreber 1775
Arctic sea birds	SBA	*Uria lomvia*, Linnaeus 1758
Boreal sea birds	SBB	*Fratercula arctica*, Linnaeus 1758
Long rough dab	LRD	*Hippoglossoides platessoides*, Fabricius 1780
Greenland halibut	GRH	*Reinhardtius hippoglossoides*, Walbaum 1792
Mackerel	MAC	*Scomber scombrus*, Linnaeus 1758
Haddock	HAD	*Melongrammus aeglefinus*, Linnaeus 1758
Saithe	SAI	*Pollachius virens*, Linnaeus 1758
Redfish	RED	Beaked redfish *Sebastes mentella*, Travin 1951
Blue whiting	BWH	*Micromesistius poutassou*, A. Risso 1827
Norwegian Spring Spawning herring	SSH	*Clupea harengus*, Linnaeus 1758
Northeast Arctic cod	NCO	*Gadus morhua*, Linnaeus 1758
Polar cod	PCO	*Boreogadus saida*, Lepechin 1774
Capelin	CAP	*Mallotus villosus*, Müller 1776
Sharks, other	SHO	*Squalus acanthias*, Linnaeus 1758
Demersals, other	DEO	Ling (*Molva molva*, Linnaeus 1758), and tusk (*Brosme brosme*, *Linnaeus 1758*)
Pelagic large	PEL	Atlantic salmon (*Salmo salar*, Linnaeus 1758)
Pelagic small	PES	Lumpsucker (*Cyclopterus lumpus*, Linnaeus 1758) and Norway pout (*Trisopterus esmarkii*, Nilsson 1855)
Redfish, other	REO	Golden redfish: *Sebastes norvegicus*, Ascanius, 1772
Demersal, other large	DEL	Monkfish (*Lophius piscatorius*, Linnaeus 1758), Atlantic halibut (*Hippoglossus hippoglossus*, Linnaeus 1758), Atlantic wolffish (Anarhichas *lupus*, Linnaeus 1758), Northern wolffish (*Anarhichas denticulatus*, Krøyer 1845) and spotted wolffish (*Anarhichas minor*, Olafsen 1772).
Flatfish, other	FLA	European plaice (*Pleuronectes platessa*, *Linnaeus (1758)*), common dab (*Limanda limanda*, *Gottsche (1835)*)
Skates and rays	SSK	Arctic skate (*Amblyraja hyberborea*, Collett, 1879), thorny skate (*Amblyraja radiate*, Donovan, 1808), sailray (*Rajella lintea*, Fries, 1838), thornback ray (*Raja clavata* Linnaeus, 1758), round skate (*Rajella fyllae* Lütken, 1887) and spinytail skate (*Bathyraja spinicauda* Jensen, 1914).
Mesopelagic fish	MES	Pearlside (*Maurolicus muelleri*, Cocco 1838) and glacier lanternfish (*Benthosema glaciale*, J.C.H. Reinardt, 1837)
Prawn	PWN	*Pandalus borealis (*Krøyer 1838)
Cephalopods	CEP	*Gonatus fabricii* (Lichtenstein, 1818)
Red king crab	KCR	*Paralithodes camtschaticus* (Tilenau, 1815)
Snow crab	SCR	*Chionoecetes opilio* (Fabricius, 1788)
Gelatineous zooplankton	ZG	*Aurelia aurita* (Linnaeus, 1758), *cyanea capillata* (Linnaeus, 1758)
Large zooplankton	ZL	*Thysanoessa inermis* (Krøyer, 1846)
Medium zooplankton	ZM	*Calanus finmarchicus* (Gunnerus, 1770)
Small zooplankton	ZS	Small copepods, oncaea, pseudocalanus, (*Oithona similis*, Claus (1866))
Dinoflagellates	DF	*Phaeocystis pouchetii* (Lagerheim, 1896) and *Emiliania huxleyii* (Lohmann)
Small phytoplankton	PS	Flagellates
Large phytoplankton	PL	Diatoms
Predatory benthos	BC	Echinoderms, sea urchins, annelids and anemones
Detrivore benthos	BD	Selected annelids, echinoderms
Benthic filter feeders	BFF	Selected molluscs, barnacles, moss animals, anemones (*Tridonta borealis*, Schumacher (1817))
Sponges	SPO	*Geodia baretti* (Bowerbank, 1858)
Corals	COR	*Lophelia pertusa* (Linnaeus, 1758)
Pelagic bacteria	PB	
Benthic bacteria	BB	
Refractory detritus	DR	
Carrion	DC	
Labile detritus	DL	

The initial values of NoBa Atlantis resemble the ecosystem situation in the early 1980s, with a cold ocean climate, low primary and secondary production, but abundant pelagic and demersal fish[29], [15]. The model was tuned to be stable and have all components within reasonable limits after a spin-up time of 25 years [10]. Here, ‘reasonable limits’ meant individual weights and numbers staying within 0.5–1.5 times the initial level when repeating oceanographic input for one year, thus excluding any impact of climatic variation. This provided a stable initial state. Also, the diets were tuned to reflect empirical knowledge [30] and to produce stable runs. The model was run without explicit fisheries pressure on the components, but the fisheries mortality was accounted for in the quadratic mortality parameter.

### Key parameters and species for perturbations

[12] identified four of the most important processes in Atlantis; growth rate, recruitment, consumption rate and quadratic mortality (hereafter; mortality) rates. The equations for these processes are given below, and the specific parameters that have been perturbed are indicated in bold.

The maximum growth rate (***mum_XXX***) rate in NoBa was calculated as the maximum weight the animal can gain per day following [28]. In NoBa, weight-at-age observations from assessment reports were used for the commercial components, whereas literature, grey literature and expert knowledge made up the basis for the non-commercial components [10]. Maximum growth rate at age was then calculated as the necessary weight gain per day to allow the individual to achieve the appropriate weight gain before moving to the next cohort.

The grazing term in NoBa was represented by the equation, again following [11]:

$$Gr_{prey}=\frac{B∙C∙B_{prey}^{*}}{1+\frac{C∙\sum_{i}\left(E_{i}∙B_{prey}^{*}\right)}{mum}}$$ (1)

Where

$$B_{prey}^{*}=p_{prey,CX}∙\delta_{overlap}∙\delta_{habitat}∙\delta_{size}∙B_{prey}$$ (2)

was the available biomass of the prey after all refuge (d) was taken into account [31]. *p*_*prey*,*CX*_ was the availability (range between 0 and 1) of the prey to the predator CX. *B* was the biomass of predator CX and *E*_*i*_ is the assimilation efficiency of the predator from the different groups. In the denominator, the growth rate of the predator, ***mum***_,_ was used.

Mortality in NoBa Atlantis was represented by:

$$M_{CX}=((mL+mQ∙{Num}_{CX}+mSt)∙{Num}_{CX})$$ (3)

where *mL* was the linear mortality term and ***mQ*** the quadratic mortality (density-dependent) term for component *CX* *[31]*. The *mSt* term included mortality due to starvation for vertebrates. *Num*_*CX*_ represented the numbers in an age group.

The recruitment in NoBa followed one of two equations [31]; either a constant recruitment per reproducing adult per year (**KDENR**):

Rc=KDENR (4)

or a modified Beverton-Holt recruitment function described by:

$$Rc=\frac{Sp∙BHa}{Biom+BHb}$$ (5)

where ***BHa*** was the Beverton-Holt alpha, *BHb* the Beverton-Holt beta, *Sp* the spawn produced and *Biom* the total biomass of the species [31].

To limit the number of runs and computational cost we only perturbed the parameters of key species and functional groups in the Barents Sea system, including 4 groups of zooplankton, 3 species of pelagic fish (capelin, herring and polar cod) and the dominant top predators Northeast Atlantic cod and minke whales. For each vertebrate species, all four key parameters (Table 2) were perturbed in the model. For zooplankton, we perturbed the same parameters except recruitment, as the invertebrate components in Atlantis represented biomass pools and did not have the same recruitment functions as the vertebrate components. Of the vertebrate key species, only minke whales used the constant recruitment (Eq 4). Polar cod, herring, capelin and cod all used the Beverton-Holt recruitment function (Eq 5), however herring, capelin and polar cod had lognormal variation added to their Beverton-Holt recruitment function.

**Table 2:** Overview of the perturbed parameters.

Parameter perturbed	Parameter description
***mum_XXX***	The maximum growth (in weight) gained per day
***BHa***	The slope in the Beverton-Holt recruitment function
***C***_*CX*_	The consumption rate of consumer CX
***mQ***_***CX***_	The quadratic mortality term for component *CX*

As the model did not include fisheries directly, the quadratic mortality term was perturbed, as this then also represented mortality due to loss through fisheries.

The parameters perturbed in this study are among those usually most heavily tuned when setting up an Atlantis model. For the NoBa model, the growth, consumption and mortality rates were initially set as close to observed values as possible [10]. However, while the same was carried out for recruitment, recruitment is typically highly variable, and thus subject to higher possible error [10].

In all runs but four, the parameters were changed +/- 25%. Mortality rate differed between juvenile and adult stages and between species. Consumption and growth rate were resolved at each age class. Thus, when varying parameters resolved at age-class stage, all age classes were equally perturbed at the same time. These parameters were first varied one-at-a-time (OAT; N = 68 runs). From these runs, we defined a selection of combined parameter perturbations based on the biomass changes of all groups in the model (N = 36 runs, further described below).

A parameter change of +/- 25% is well within observed natural levels of variation (e.g. [14, 22, 32]. It has to be emphasized that the natural levels of variation are realized levels and cannot be directly compared to the changes applied on the parameters. The scope of this sensitivity analysis was to identify where the model response to a given perturbation is particularly strong, hence the actual size of the perturbation may be less relevant. However, to test how the model responded to extreme perturbations, we mimicked some extremes in observed values (e.g.[14, 22]: i) Increasing herring recruitment by 230%; ii) decreasing herring recruitment by 90%; iii) increasing growth rate of cod by 70% and iv) decreasing growth rate of cod by 39%. The differences in herring recruitment was calculated based on Fig 9 in [22], where numbers of recruits for the period 1981–1996 was extracted, and the average of the timeseries, the minimum and maximum year was used to find the two extremes. The changes to the growth rates of cod were calculated based on the annual weight gain of cod in Fig 8 in [14]. Finally, one control run was performed with no parameter variations. All simulations were initiated at the same biomass levels and experienced the same number of years in the spin up period.

All experiments were run for a length of 55 years with temperature, salinity and volume fluxes for one year (1981) looped for the whole simulation period to maintain a stable environment throughout the experiments. This particular year represents a cold year in the system. Thus, the simulation experiments were not expected to reproduce the observed development of the ecosystems and its components, as these had gone through a consistent warming impacting both ecosystem structure and function[33, 34, 35]. The ability to reproduce the ecosystem development over time will be the focus of a separate skill assessment (following among other the methods of[6]. For each run, the average biomasses over the last 10 years of the simulations were calculated. A spin-up time of 25 years was used [10] and the model output was stable for roughly 20 years before the results were extracted.

To assess the effects of parameter perturbations, we calculated an *impact factor* for each run according to the magnitude of the change in absolute biomass of the key species compared to the control run. The impact factor was calculated by first splitting the changes in the average biomass of each species into four categories: no-impact, low-impact, medium-impact and high-impact, depending on the magnitude of the biomass change. These four categories were given a weight accordingly to the category (Table 3). The impact factor was then calculated as the mean of the weights of all species within each simulation.

**Table 3:** Categories of runs according to the absolute change in biomass compared to the control run.

Absolute change in biomass	Category	Impact factor
0–5%	No impact	1
5–10%	Low impact	2
10–20%	Medium impact	3
>20%	High impact	4

For the combination runs, the runs were paired based on their impact factor. High-impact runs were paired with both high-impact and low-impact runs, and low-impact runs were paired with both high-impact and low-impact runs from each group of parameters (Table 4). The reasoning behind the pairing of parameter settings was to evaluate possible additive and non-additive effects that would evolve from these combinations.

**Table 4:** Overview of runs and parameter changes.

Sim. name	Description	Parameter pert.	Original value	Change
**Run 00**	Control run	-	**-**	**-**
**Run 01**	Increase growth rate for cod	mum_NCO	160, 54, 102, 180, 214, 228, 228, 264, 270, 270	25%
**Run 02**	Decrease growth rate for cod	mum_NCO	160 .0, 54.0, 102.0, 180.0, 214.0, 228.0, 228.0, 264.0, 270.0, 270.0	-25%
**Run 03**	Increase growth rate for herring	mum_SSH	1.20, 1.80, 2.90, 1.40, 1.80, 1.10, 0.80, 0.60, 0.80, 0.20	25%
**Run 04**	Decrease growth rate for herring	mum_SSH	1.20, 1.80, 2.90, 1.40, 1.80, 1.10, 0.80, 0.60, 0.80, 0.20	-25%
**Run 05**	Increase growth rate for polar cod	mum_PCO	2.40, 0.60, 0.45, 0.20, 0.20, 0.20, 0.20, 0.20, 0.40, 0.40	25%
**Run 06**	Decrease growth rate for polar cod	mum_PCO	2.40, 0.60, 0.45, 0.20, 0.20, 0.20, 0.20, 0.20, 0.40, 0.40	-25%
**Run 07**	Increase growth rate for capelin	mum_CAP	0.33, 0.25, 0.27, 0.28, 0.28	25%
**Run 08**	Decrease growth rate for capelin	mum_CAP	0.33, 0.25, 0.27, 0.28, 0.28	-25%
**Run 09**	Increase growth rate for minke whales	mum_MWH	1·10^5^, 2·10^4^, 2·10^4^, 2·10^4^, 2·10^4^, 2·10^4^, 8·10^3^, 8·10^3^, 8·10^3^, 8·10^3^	25%
**Run 10**	Decrease growth rate for minke whales	mum_MWH	1·10^5^, 2·10^4^, 2·10^4^, 2·10^4^, 2·10^4^, 2·10^4^, 0.8·10^4^, 0.8·10^4^, 0.8·10^4^, 0.8·10^4^	-25%
**Run 11**	Decrease growth of large zooplankton	Mum_ZL	0.076	-25%
**Run 12**	Decrease growth of medium zooplankton	Mum_ZM	0.1	-25%
**Run 13**	Decrease growth of small zooplankto	Mum_ZS	3.55	-25%
**Run 14**	Decrease growth of gelatineous zooplankton	Mum_ZG	0.02	-25%
**Run 15**	Increase growth of large zooplankton	Mum_ZL	0.076	25%
**Run 16**	Increase growth of medium zooplankton	Mum_ZM	0.1	25%
**Run 17**	Increase growth of small zooplankton	Mum_ZS	3.55	25%
**Run 18**	Increase growth of gelatineous zooplankton	Mum_ZG	0.02	25%
**Run 19**	Decrease cod consumption	C_NCO	90, 110, 250, 400, 550, 1250, 1600, 1900, 2000, 2300	-25%
**Run 20**	Increase cod consumption	C_NCO	90, 110, 250, 400, 550, 1250, 1600, 1900, 2000, 2300	25%
**Run 21**	Increase herring consumption	C_SSH	2.8, 6.9, 13.4, 16.5, 20.4, 22.9. 24.6, 25.98, 27.90, 28.2	25%
**Run 22**	Decrease herring consumption	C_SSH	2.8, 6.9, 13.4, 16.5, 20.4, 22.9, 24.6, 25.98, 27.90, 28.2	-25%
**Run 23**	Increase capelin consumption	C_CAP	1.5, 3, 4, 6, 8	25%
**Run 24**	Decrease capelin consumption	C_CAP	1.5, 3, 4, 6, 8	-25%
**Run 25**	Increase polar cod consumption	C_PCO	3, 1.3, 2.03, 2.6, 2.5, 2.7, 3.2, 3.9, 4.8, 5.6	25%
**Run 26**	Decrease polar cod consumption	C_PCO	3, 1.3, 2.03, 2.6, 2.5, 2.7, 3.2, 3.9, 4.8, 5.6	-25%
**Run 27**	Increase minke whale consumption	C_MWH	7.9·10^5^, 8.9·10^5^, 1·10^6^, 1.1·10^6^, 1.2·10^6^, 1.3·10^6^, 1.4·10^6^, 1.4·10^6^, 1.5·10^6^, 1.5·10^6^	25%
**Run 28**	Decrease minke whale consumption	C_MWH	7.9·10^5^, 8.9·10^5^, 1·10^6^, 1.1·10^6^, 1.2·10^6^, 1.3·10^6^, 1.4·10^6^, 1.4·10^6^, 1.5·10^6^ 1.5·10^6^	-25%
**Run 29**	Decrease consumption by large zooplankton	C_ZL	0.2	-25%
**Run 30**	Decrease consumption by medium zooplankton	C_ZM	0.4	-25%
**Run 31**	Decrease consumption by small zooplankton	C_ZS	0.4	-25%
**Run 32**	Decrease consumption by gelatineous zooplankton	C_ZG	0.5	-25%
**Run 33**	Increase consumption by large zooplankton	C_ZL	0.2	25%
**Run 34**	Increase consumption by medium zooplankton	C_ZM	0.4	25%
**Run 35**	Increase consumption by small zooplankton	C_ZS	0.4	25%
**Run 36**	Increase consumption by gelatineous zooplankton	C_ZG	0.5	25%
**Run 37**	Increase cod recruitment	Bhalpha_NCO	3.00·10^11^	25%
**Run 38**	Decrease cod recruitment	Bhalpha_NCO	3.00·10^11^	-25%
**Run 39**	Increase capelin recruitment	Bhalpha_CAP	1.50·10^10^	25%
**Run 40**	Decrease capelin recruitment	Bhalpha_CAP	1.50·10^10^	-25%
**Run 41**	Increase herring recruitment	Bhalpha_SSH	7.00·10^7^	25%
**Run 42**	Decrease herring recruitment	Bhalpha_SSH	7.00·10^7^	-25%
**Run 43**	Increase polar cod recuritment	Bhalpha_PCO	4.30·10^8^	25%
**Run 44**	Decrease polar cod recruitment	Bhalpha_PCO	4.30·10^8^	-25%
**Run 45**	Increase minke whale recruitment	KDENR_MWH	0.45	25%
**Run 46**	Decrease minke whale recruitment	KDENR_MWH	0.45	-25%
**Run 47**	Increase cod mortality	jmQ_NCO/ mQ_NCO	1.48·10^−9^, 5.48·10^−9^	25%
**Run 48**	Decrease cod mortality	jmQ_NCO/ mQ_NCO	1.48·10^−10^, 5.48·10^−9^	-25%
**Run 49**	Increase herring mortality	jmQ_SSH/ mQ_SSH	2.47·10^−13^, 2.11·10^−13^	25%
**Run 50**	Decrease herring mortality	jmQ_SSH/ mQ_SSH	2.47·10^−13^, 2.11·10^−13^	-25%
**Run 51**	Increase capelin mortality	jmQ_CAP/ mQ_CAP	1.48·10^−18^, 1.1·10^−22^	25%
**Run 52**	Decrease capelin mortality	jmQ_CAP/ mQ_CAP	1.48·10^−18^, 1.1·10^−22^	-25%
**Run 53**	Increase polar cod mortality	jmQ_PCO/ mQ_PCO	1.48·10^−13^, 5.48·10^−13^	25%
**Run 54**	Decrease polar cod mortality	jmQ_PCO/ mQ_PCO	1.48·10^−13^, 5.48·10^−13^	-25%
**Run 55**	Increase minke whale mortality	jmQ_MWH/mQ_MWH	9.49·10^−7^, 7.48·10^−8^	25%
**Run 56**	Decrease minke whale mortality	jmQ_MWH/mQ_MWH	9.49·10^−7^, 7.48·10^−8^	-25%
**Run 57**	Decrease mortality of large zooplankton	mQ_ZL	8.00·10^−10^	-25%
**Run 58**	Decrease mortality of medium zooplankton	mQ_ZM	2.50·10^−8^	-25%
**Run 59**	Decrease mortality of small zooplankton	mQ_ZS	6.00·10^−8^	-25%
**Run 60**	Decrease mortality of gelatineous zooplankton	mQ_ZG	4.50·10^−8^	-25%
**Run 61**	Increase mortality of large zooplankton	mQ_ZL	8.00·10^−10^	25%
**Run 62**	Increase mortality of medium zooplankton	mQ_ZM	2.50·10^−8^	25%
**Run 63**	Increase mortality of small zooplankton	mQ_ZS	6.00·10^−8^	25%
**Run 64**	Increase mortality of gelatineous zooplankton	mQ_ZG	4.50·10^−8^	25%
**Run 65**	Increase growth rate of cod	Mum_NCO	160, 54, 102, 180, 214, 228, 228, 264, 270, 270	70%
**Run 66**	Increase recruitment of herring	BHalpha_SSH	7.00·10^7^	230%
**Run 67**	Decrease growth rate of cod	Mum_NCO	160, 54, 102, 180, 214, 228, 228, 264, 270, 270	39%
**Run 68**	Decrease recruitment of herring	BHalpha_SSH	7.00·10^7^	90%
**Run 69**	Decrease consumption and mortality for cod	Run 19 + Run 48		
**Run 70**	Decrease consumption for cod + decrease recruitment of capelin	Run 19 + Run 40		
**Run 71**	Decrease recruitment of capelin and herring	Run 40 + Run 42		
**Run 72**	Decrease minke whale mortality + decrease capelin growth	Run 56 + Run 8		
**Run 73**	Decrease consumption of capelin + decrease mortality of cod	Run 24 + Run 48		
**Run 74**	Decrease growth rate of polar cod and of capelin	Run 6 + Run 8		
**Run 75**	Decrease growth rate of minke whales + decrease consumption for cod	Run 10 + Run 19		
**Run 76**	Decrease mortality of cod + decrease recruitment of capelin and herring	Run 48 + Run 71		
**Run 77**	Increase C_ZS + decrease mum_ZL	Run 35 + run 11		
**Run 78**	Increase C_ZS+ decrease bhalpha_MHW	Run 35 + run 46		
**Run 79**	Increase C_ZS + increase mQ_NCO	Run 35 + run 47		
**Run 80**	Decrease mum_ZL+ decrease Bhalpha_MWH	Run 11+ run 46		
**Run 81**	Decrease mum_ZL+increase mQ_NCO	Run 11 + run 47		
**Run 82**	Decrease Bhalpha_MWH+ increase mQ_NCO	Run 36 + run 35		
**Run 83**	Decrease mum_SSH+increase C_ZS	Run 4 + run 35		
**Run 84**	Decrease mum_SSH+ decrease Bhalpha_MWH	Run 4 + run 46		
**Run 85**	Decrease mum_SSH+decrease mum_ZL	Run 4 + run 11		
**Run 86**	Decrease mum_SSH+increase mQ_NCO	Run 4 + run 47		
**Run 87**	Increase C_PCO+increase C_ZS	Run 25 + run 35		
**Run 88**	Increase C_PCO+ decrease Bhalpha_MWH	Run 25 + run 46		
**Run 89**	Increase C_PCO+decrease mum_ZL	Run 25 + run 11		
**Run 90**	Increase C_PCO+increase mQ_NCO	Run 25 + run 47		
**Run 91**	Increase Bhalpha_CAP+increase C_ZS	Run 39 + run 35		
**Run 92**	Increase Bhalpha_CAP+decrease Bhalpha_MWH	Run 39 + run 46		
**Run 93**	Increase_Bhalpha_CAP+decrease mum_ZL	Run 39 + run 11		
**Run 94**	Increase Bhalpha_CAP+increase mQ_NCO	Run 39 + run 47		
**Run 95**	Decrease mQ_PCO+increase C_ZS	Run 54 + run 35		
**Run 96**	Decrease mQ_PCO+decrease Bhalpha_MWH	Run 54 + run 46		
**Run 97**	Decrease mQ_PCO+decrease mum_ZL	Run 54 + run 11		
**Run 98**	Decrease mQ_PCO+increase mQ_NCO	Run 54 + run 47		
**Run 99**	Increase C_PCO+decrease mum_SSH	Run 25 + run 4		
**Run 100**	Increase C_PCO+ increase Bhalpha_CAP	Run 25 + run 39		
**Run 101**	Increase C_PCO+decrease mQ_PCO	Run 25 + run 54		
**Run 102**	Decrease mum_SSH+ increase Bhalpha_CAP	Run 4 + run 39		
**Run 103**	Decrease mum_SSH+decrease mQ_PCO	Run 4 + run 54		
**Run 104**	Increase Bhalpha_CAP+decrease mQ_PCO	Run 39 + run 54		

In addition to these 28 combination runs (run 77–104, Table 4), 8 runs with different combinations of high-impact and low-impact on the key species alone were performed (run 69–76, Table 4). In these 8 runs exclusively, the response in biomass for the key species was taken into account when pairing the parameter settings.

To assess whether the responses to combined parameter perturbations were additive or non-additive, we followed the approach used by [36]. A species response was considered additive if the biomass change of the component in the combined run equalled (within ± 1%) the sum of biomass change when the parameters were perturbed separately, i.e.

$$\Delta P_{1}P_{2}–(\Delta P_{1}+\Delta P_{2})<0.01$$ (6)

Here, *ΔP*_x_ represented biomass changes for a specific component in one of the OAT runs compared to the control run, whereas *ΔP*_*1*_*P*_*2*_ represented biomass changes for the same component in the combined simulation compared to the control run. Responses were defined as non-additive if the biomass change differed (more than ± 1%) from the pair of single perturbed parameter runs, i.e.

$$\Delta P_{1}P_{2}–(\Delta P_{1}+\Delta P_{2})>0.01$$ (7)

For the additive and non-additive effects, key species as well as all other components (mammals, birds, fish, lower trophic levels, benthos and bacteria) were examined. The non-additive effects were furthermore identified as either *antagonistic* or *synergistic*, depending on the biomass change for the combined run being smaller or higher than the sum of biomass change, when parameters were perturbed separately, respectively:

$$abs(\Delta P_{1}P_{2})<abs(\Delta P_{1}+\Delta P_{2})$$ (8)

$$abs(\Delta P_{1}P_{2})>abs(\Delta P_{1}+\Delta P_{2})$$ (9)

All calculations and figures were produced with R (R Development Core Team, 2008).

## Results

In total, 104 simulations were performed. In 66 runs, one or more of the key species/groups experienced changes in biomass > 20%. On the other end, only 3 simulations gave responses in the key species/groups below 5%. Of the 66 runs with the strongest impact on the key species/groups (i.e., one or more species/groups with biomass change > 20%), 39% were combined runs, and 42% included perturbations on one of the zooplankton components. The 15 runs with the highest impact factor all included parameter perturbations on the zooplankton level.

### System impact of parameter perturbations

**Fig 2:**
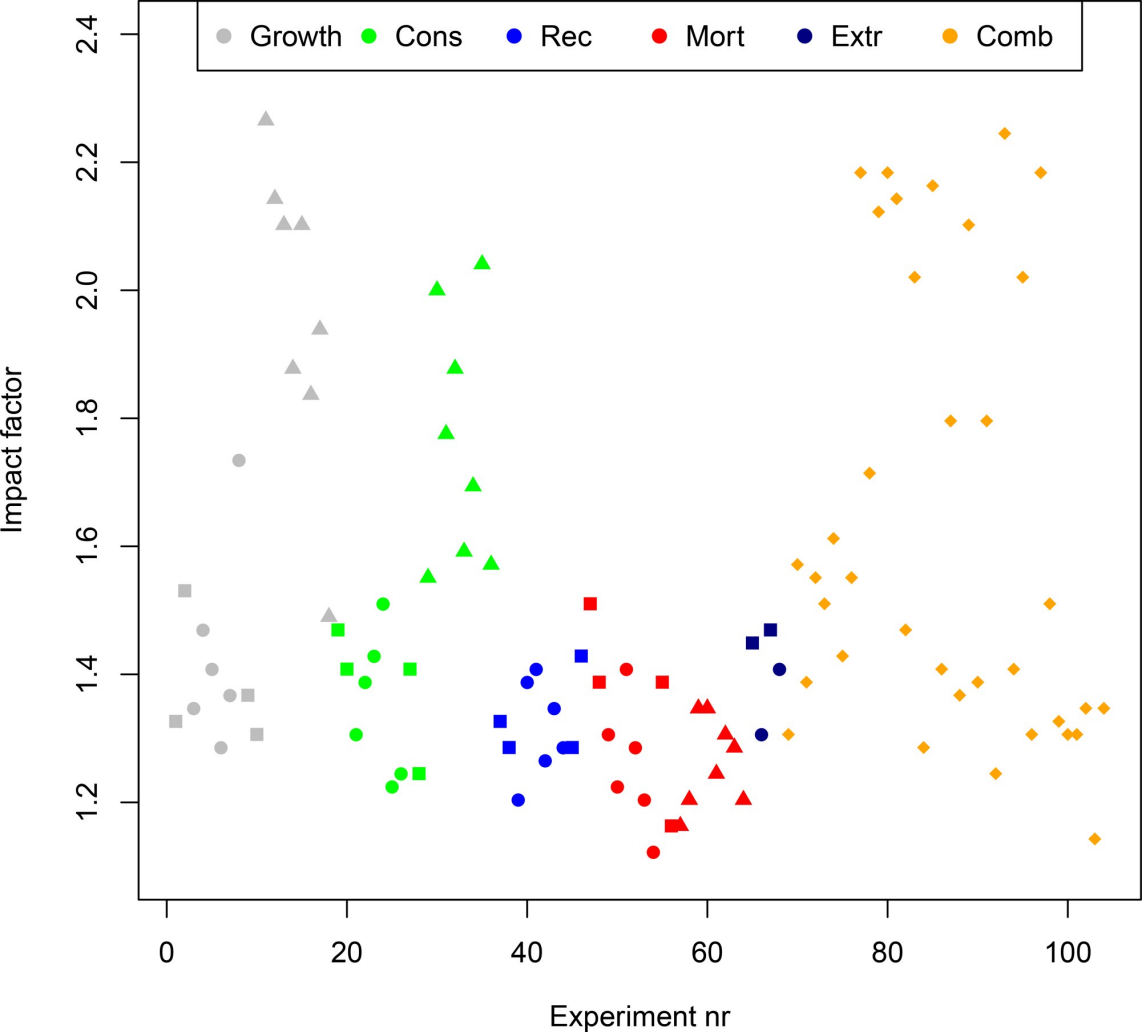
Impact factor. The figure shows the impact factor of all the simulations. The impact factor indicates how many species experience a change in the biomass and is weighted according to the magnitude of the impact. The experiments are color-coded according to which parameter is tuned, where grey is growth rate (Growth), green is consumption rate (Cons), red is mortality rate (Mort), blue is recruitment (Rec), dark blue are the extreme (Extr) parameter changes, and orange represents combination runs (Comb). In addition, the shape of the dot indicates which trophic level that has been perturbed. Triangles represents perturbations at zooplankton level, squares at top predator level, circles at mid-level and diamonds represents combined runs.

The importance of each parameter was assessed based on the impact factor for each simulation (Fig 2). The single parameter change that gave the lowest response in the ecosystem, was decreasing the polar cod mortality (Fig 2: the lowest red dot). Decreasing the growth rate parameter of large zooplankton gave the highest response in the ecosystem (Fig 2: uppermost grey triangle). Mortality and recruitment parameter perturbations (Fig 2; red and blue marks) had the lowest impact on the system and gave low response rates for a large number of model components. Perturbing consumption rate for the top and mid-trophic level species (green squares and circles) gave small responses, hence low impact factors. However, perturbing the consumption rate for the zooplankton (green triangles) resulted in changing biomasses for several components, causing a higher impact factor at the system level. Perturbing the growth rate parameter had, as for the consumption rates, a larger impact when performed at the zooplankton level (Fig 2; grey triangles), compared to at mid- and top-trophic levels (Fig 2; grey squares and circles). The first 8 combined parameter perturbations (Fig 2; orange diamonds), including different combinations of perturbing mortality of cod, recruitment of capelin and growth rate parameter of minke whales, resulted in intermediate effects on top and mid-trophic level key species. The last 28 combination runs (Fig 2; orange diamonds) were parameter combinations that represented simulations with both low and high impact factor for a particular parameter (Table 4). The strongest responses were associated with perturbing the consumption rate of small zooplankton and growth rate parameter of large zooplankton. Of the 15 runs that gave the strongest impacts, 10 were combined runs, all including at least one perturbation on a zooplankton level.

### Species responses and sensitivity to parameter perturbations

**Fig 3:**
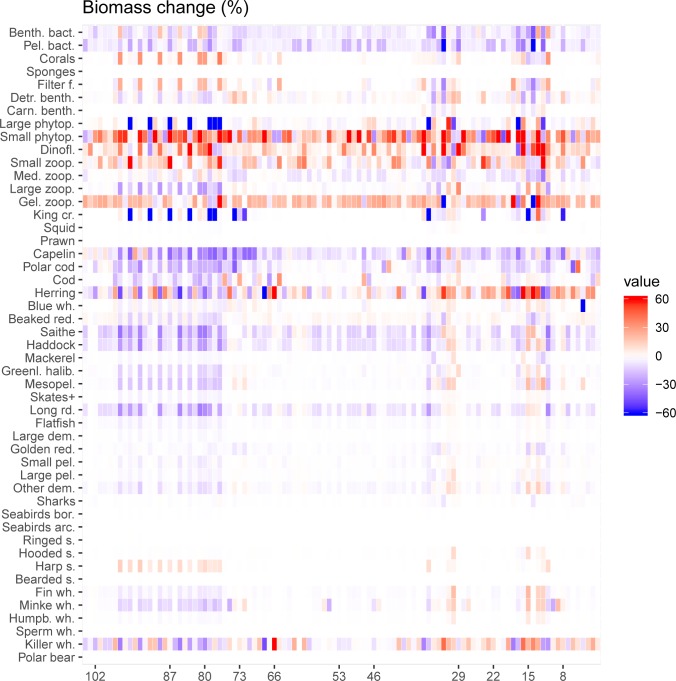
Component response to perturbations. Biomass change for all species in all runs. Dark red/dark blue areas are changes above or below 60%. While fish and marine mammals experience a negative response to a majority of the simulations, the opposite is found in the zooplankton, due to predator-prey interactions. Harp seal (*Pagophilus groenlandicus*) is one of a few species to have mostly positive responses (although low).

There were large variations among species and groups in strength of responses to the different perturbations, and thus how sensitive the species and groups were to the parameterization of the key species/groups in the model system. Fig 3 shows the biomass changes in all species/groups across the 104 runs. Marine mammals and seabirds generally responded weakly to parameter perturbations, except for killer whales who showed relatively large biomass changes (> ± 20%) in 25 runs. The killer whale responses, which were all indirect responses as the parametrization of killer whales were not perturbed, were also stronger than the responses in minke whales, which were directly perturbed. Among the fish, the strongest responses were found in the pelagic fish; capelin, polar cod and herring. Of the non-perturbed fish components, there were large changes in saithe, haddock and long rough dab; with the responses of these three species having a correlation >0.86. These fish frequently experienced biomass changes >20%.

The non-perturbed lower trophic levels generally experienced a stronger change in average biomass in a larger fraction of the runs compared to those seen in higher trophic levels. Among these, squid, prawns, carnivore benthos and sponges showed the weakest responses to the perturbations, whereas phytoplankton, red king crab and bacteria had the strongest responses to the perturbations. The strong responses seen in red king crab and bacteria is explained by predator-prey relationship between these two components and the zooplankton components.

The perturbations to the zooplankton level in isolation or in combination caused high impact factors (Fig 2; triangles for OAT perturbations). The high effects of these perturbations trickled through almost other all components of the foodweb at all levels in the system.

### Additive and non-additive effects

Whether responses to combined perturbations were additive or non-additive depended to a large degree on trophic level of the component (Table 5). The additive responses were most frequent at the top-predator level, whereas the lower trophic levels were more associated with non-additive responses (Table 5). Phytoplankton and zooplankton experienced a higher number of synergistic effects (i.e. stronger response than expected from single parameter perturbations) compared to other trophic levels. Splitting the lower trophic levels into phytoplankton and zooplankton showed a slightly higher number of additive runs in phytoplankton (11.2% compared to 5.6%), whereas zooplankton had a higher fraction of synergistic runs (72.2% compared to 47.2%). Species only experiencing indirect effects due to parameter perturbations had a lower fraction of synergistic responses than the species that were perturbed directly. The synergistic effects were not large (<15%).

**Table 5:** Additive/Non-additive effects on component groups (in % of runs). All 36 combination runs were considered.

	Mammals	Birds	Fish	Lower troph. lev.	Benthos	Bacteria
**Additive**	55.6	100	25	11.1	72.2	2.8
**Synergistic**	16.7	0	5.6	61.1	13.9	19.4
**Antagonistic**	27.8	0	69.4	27.8	13.9	77.8

Top predators experienced a higher number of additive effects than the species at mid-trophic levels. Capelin and herring experienced opposite effects; where capelin in most cases responded to combined perturbations with antagonistic effects, dampening the responses, herring experienced by far the most synergistic effects of perturbing the key components (Table 6).

**Table 6:** Overview of additive/non-additive effects (in % of runs) on the key species in the 36 combination runs.

	Cod	Herring	Polar cod	Minke whales	Capelin	Large z.p	Medium z.p	Small z.p	Gel. z.p
**Additive**	58.3	0	16.7	72.2	2.8	22.2	19.4	2.8	2.8
**Synergistic**	19.4	55.6	33.3	0	25	41.7	27.8	61.1	72.2
**Antagonistic**	22.2	44.4	50.0	27.8	72.2	36.1	52.8	36.1	25

### Model performance

**Fig 4:**
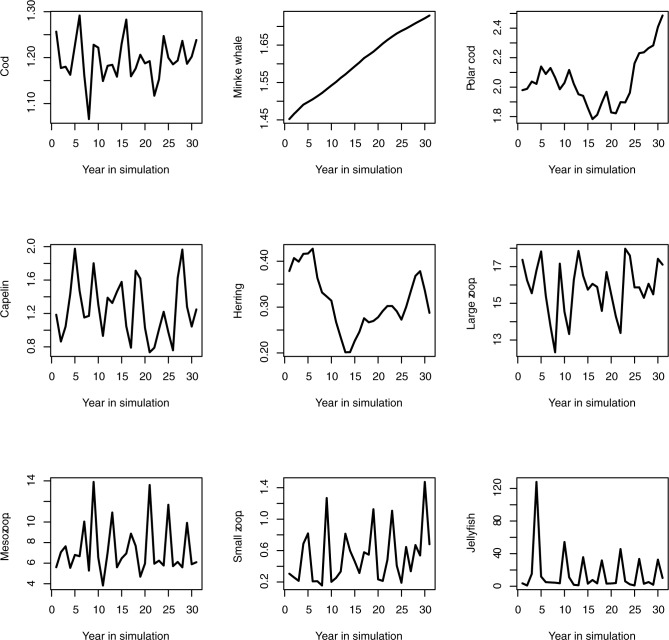
Development of biomass in key species. Relative change in biomass of key species in control run compared to initial values.

The model was run with no variability in physics or fisheries pressure, which limits the possibilities to compare the model output to the temporal development of the ‘real world’. However, one rule-of-thumb that has been applied in several Atlantis models (e.g. [37] is that the components should remain within 0.5–1.5 of the initial biomass levels after the burn-in period. The relative biomasses of the 9 key species from the control run are shown in Fig 4. Over the simulation time, cod, capelin and small zooplankton stayed within 50 and 150% of the initial biomass level. Minke whales and polar cod showed a consistent slow increase, whereas herring decreased. The large and medium zooplankton stabilised at levels 8–16 times larger than the initial biomass levels, whereas jellyfish oscillated between 1–50 times initial levels. Hence, the biomass levels for the zooplankton components were on the higher end compared to initial values. However, the biomass estimates at these levels are both uncertain and highly variable, so we do not consider this as a critical issue. We also assessed individual weights to ensure realistic body sizes. Of all cohorts for the vertebrate components in the model, 75% were within 0.5–1.5 of their initial weights, and we found this satisfactory. Furthermore, timing of phytoplankton and zooplankton blooms were within what has been observed. Predation was compared to observations and literature and corrected for those species that either had unrealistic diets or preyed heavily on a single component, causing this to decrease.

In summary, we conclude that these comparisons indicate that the majority of the components stayed within reasonable levels and hence that this version of the model was in a sufficient state to be applied for sensitivity analysis. For other applications, e.g. management strategy evaluations, we conclude that the model needs further tuning and refinement.

## Discussion and conclusions

End-to-end models, such as Atlantis, have become increasingly important in ecosystem studies and in development and testing of ecosystem-based management strategies [38]. These models should not be used in tactical decisions such as defining quotas [39], but at the same time they are important tools for strategic planning and shifting management approaches towards ecosystem-based fisheries management[8]. They can also be actively used in integrated ecosystem assessments, e.g. through management strategy evaluations[8,40]. Before taking such steps, however, the model responses, sensitivity and uncertainty should be evaluated. This will increase credibility and trust in the model, managers and other users will have a transparent assessment of the reliability and uncertainty associated with the model projections. We have therefore run this initial sensitivity analysis on core components of the ecosystem foodweb in the Norwegian and Barents seas, which are known to strongly interact.

The four key variables perturbed in this study; growth rate, mortality rate, recruitment and consumption rate, are generally the most sensitive variables in Atlantis [12]. When calculating the realized growth rate for cod from Fig 8 in [14], this indicate that annual variation in the maximum growth rate parameter (*mum*) may range from -39 to 71%. Therefore, we considered the applied change of +/- 25% as used in our perturbations as moderate. Nevertheless, when applying the extreme values reported by [14] and by [22] regarding herring, we found that the size of the perturbations was not reflected in the model results. This indicates a strong buffering capacity within the model system. In the real system, the difference between the cascading effects of the first capelin collapse, when also the polar cod and the herring biomasses were low, and the ecosystem responses in the following two capelin collapses [14] when the two other stocks were at higher levels, were striking. It is possible that also in the NoBa system the cumulative effects of several key stocks increasing/decreasing in concert will have a much stronger ecosystem response than what can be seen from changes in a single stock. The system vulnerabilities to cumulative changes across stocks should be further investigated in the modelled system.

The empirical information available differ between the 4 perturbed parameters. Typically, information on weight at age used to calculate the growth rate parameter is available for all commercially exploited stocks through the ICES stock assessment working group reports (e.g. [41, 42]. There was, however, less information and more uncertainty regarding the growth of the youngest age classes. Recruitment, consumption and mortality rates in the real system are both more variable and more difficult to estimate than growth rates (e.g. [43]. In the NoBa model these parameters were, as far as possible, based on empirical knowledge. Still, considerable tuning was required to reach model equilibrium [10]. In particular, the mortality rate in the model turned out to be much lower than the mortality rate suggested in ICES working group reports [10]. The reason for this was that the predation mortality is separated from the other mortality rates in the Atlantis framework. The consumption rates were initially defined based on an assumption about stomach fullness and the relationship between the weight of the stomach and the total body weight [10]. However, a substantial amount of tuning was necessary to obtain the individual weights within reasonable limits. These examples demonstrate some of the challenges in parameterizing ecosystem model; both finding but also reformulating empirical observations to parameter values. Furthermore, they also demonstrate the challenge of comparing parameter values to empirical values, as the parameter values and the process not necessarily can be directly compared.

### System impact

At the system level, the responses following the zooplankton perturbations show that there are strong bottom-up dependencies within this ecosystem model. [12] and [44] found similar dependencies in other Atlantis models which had been developed for ecosystem types very different to that in NoBa. This could therefore indicate the behaviour is a feature of the Atlantis formulations. However, in the real world ecosystem of the Barents Sea, [16] pointed out that the system structure fluctuates between bottom-up and top-down, while [45] defined the system as a wasp-waist system. Either way, the large fish stocks in the Barents Sea depend on the important production from the zooplankton, although the links might potentially be stronger in the model than in the real world.

[12] identified mortality and recruitment perturbations among the four most important parameters. We found that in NoBa these two resulted in the weakest responses in the system among the four key parameters explored in the study. However, the (background non-predation) mortality used in the model was extremely low for most of these species. A 25% increase will therefore still be a low additional mortality. The reason for these low parameter values are that the predation mortality is accounted for directly in the grazing terms and this is much larger than the quadratic mortality rate. The mortality rates represented by mQ is typically low in most Atlantis models. The recruitment parameter had high values, but also high variability and a large buffering capacity (among other due to predation mortality on the juveniles). Possibly, parameter changes >25% are needed to see any effects of these parameters due to their natural high variability. The number of fish larvae entering the model system was highly variable and allowed for strong year classes. Hence, an initial parameter change of 25% on recruitment may not be expected to have the same impact as changes for the other parameters.

Overall, buffering effects were found within the system, especially when moving from lower to higher trophic levels. Buffering effects have been observed in other Atlantis models [12] and in relation to sensitivity to changes in fisheries in multispecies models [46]. It is, to some degree, also seen in the real world, where the lower trophic levels experience seasonal differences, for instance the strong seasonal spring bloom seen in northern areas. These are followed by zooplankton grazing on the phytoplankton[47]. Moving up to higher trophic levels, the amplitudes gradually weakens, the same effects are seen in the Atlantis framework.

The strong bottom-up control might change drastically when the fisheries and climate are added to scenarios, but it suggests that the lower trophic levels should be part of the added variability to the model system when testing management strategy evaluations. The systems dependency on zooplankton biomass compared to zooplankton growth should also be evaluated in future studies exploring harvest control rules. Another important aspect is the predator-prey interaction and the importance of these links when defining the outcome of changes in management. This is information not usually identified through the traditional single-stock assessment models.

### Species responses and sensitivity to parameter perturbations

The trophic level of the species being investigated, to a large degree, determined the outcome of the response to the perturbations. There was only one exception; killer whales. Their strong coupling to herring, both in terms of preference within the diet matrix and their horizontal distribution, made them very dependent on the herring prey. Although killer whales have traditionally been known to follow the herring distribution, studies have shown that they also follow the mackerel distribution during summer feeding[48]. There is an overlap between these two distributions within the NoBa domain [10]). However, a stronger dependency on the mackerel within the diet of killer whales is something that should be considered further in a skill assessment of the NoBa model. Further research on predation patterns of killer whales and how they potentially switch between these two important pelagic stocks would also benefit both the model development and the ecosystem knowledge.

Somewhat surprisingly, we noticed a strong and very similar response to the perturbations across haddock, saithe and long rough dab. These species do not have the same distributions, nor the same predator preferences [10]. However, when investigating further, these correlations in their responses are caused by a strong link to large zooplankton rather than any direct interactions between the species. The strong response of these three fish species to perturbations in large zooplankton is likely due to strong dependency of their younger cohorts on zooplankton prey. Haddock is known to prey heavily on crustaceans, whereas saithe on the other side prefers a diet consisting of more fish [49]. Not being one of the commercial species, less is known about the long rough dab and its diet preferences. However, in the North Sea, the long rough dab is known to prey mainly on crustaceans [50]. The feeding interactions of components in the system representing relatively large biomasses is an important aspect of ecosystem-based management; how sensitive is the outcome of the management advice to changing predator-prey interaction? Here, more information on non-commercial key players would be extremely useful.

Jellyfish in the system respond strongly to several parameter perturbations, due to a combination of factors. They have strong blooms and fast response rates. In addition, they are placed at approximately the same trophic level as herring and other pelagic fish [51], and are as vulnerable to changes. Their importance and role in the ecosystems are interesting, as they overlap to a large degree in prey items and distributions with the forage fish [52], and they are significant components in the Barents Sea ecosystem in terms of biomass[52,53]. [53] estimated changes in the jellyfish biomass from 19x10^6^ kg to 4906x10^6^ kg, a > 250 fold increase in the period 1980–2010, a change much larger than the jellyfish responses within our sensitivity study (and one likely to be discounted as an implausible model result if the empirical observations did not exist). However, investigating further the role and response of jellyfish to drivers such as climate change and harvesting, could highlight areas where further research is needed to provide answers on their importance for ecosystem structure and function.

Both capelin and herring were sensitive to almost any perturbation in the model. The high vulnerability of these species is likely linked to their trophic position, as small pelagic fish typically have strong variability in their population dynamics (e.g. [54]. Capelin was eaten by a wide range of species (including herring). Herring was in a similar position in the food web and also connected to capelin through the predation by herring juveniles on capelin recruits. The importance of the overlap between the two for the population development of both stocks should be further explored in future studies. It is notable that the impact on polar cod was less than for other species at the corresponding trophic level. This can be explained by the degree of horizontal overlap in the system, as the polar cod are located in the northern part of the Barents Sea with less horizontal overlap and hence less trophic connections to the other dominating species being affected (e.g. [55]). They were therefore more robust to perturbations in the rest of the system, demonstrating the importance of species distributions and food web compartmentalization in terms of food-web cascading.

Similarly,[56] using the GADGET multispecies model for the Barents Sea found relatively strong responses in capelin to changes in trophic interactions and fisheries, while herring responses were weaker. Furthermore, they also found strong responses and fluctuations at the top predator level (cod), which we did not observe in NoBa. These differences could be caused by the structural differences between the GADGET model and the Atlantis model system, and/or the fact that this version of NoBa is run without any external stressors like fisheries and climate variability. These differences should be explored further in ensemble runs with NoBa and GADGET (e.g. [57]).

### Additive and non-additive effects

The combined runs revealed both additive and non-additive effects in the model system. The additive effects were predominant in mammals and seabirds, while there was a higher occurrence of non-additive effects in fish and lower trophic levels. The number of prey-predator links are higher within these levels, as was (for most species) the degree of spatial overlap. Most of the non-additive effects were antagonistic, hence dampening the effects of combined parameter perturbations relative to perturbing the parameters in separate runs. However, synergistic effects were mostly observed in the lower trophic levels. Compared to [36], who used the Northeast U.S. Atlantis model system to explore the effects of climate and fisheries, we experience much higher numbers of non-additive effects. This might be an effect of running the model without one of the strongest drivers; the fisheries, likely increasing the relative strengths of trophic interactions and this should be kept in mind in future ‘what if’ scenarios where fisheries will be included. When testing suggested changes in harvest control rules as part of a management strategy evaluation, the dependency on the variability of the lower trophic levels should be included. From a management perspective, as [36] also argue, the non-additive effects may be crucially important. As more drivers are included, the combination of several stressors and more interactions can potentially lead to stronger synergistic effects. The high number of interactions possibly also explains the higher number of non-linear effects in the lower trophic levels. Due to the grouping into larger functional groups, these components have a much higher number of individual connections to predator species compared to the real-world system, which could introduce higher vulnerability to changes than observed in the NoBa model system.

## Conclusions

Without important drivers such as fisheries and climate, it is a challenge to evaluate the model performance. Furthermore, such comprehensive ecosystem models will never be correct for every component of the system and are associated with large uncertainties that make them inadequate for e.g. tactical decisions on harvesting quotas [39]. However, most of the components were stable throughout the simulations. Furthermore, both the biomasses and the physical environment represent a cold and low-biomass state of the Barents Sea [34], hence a state where species interactions appeared to be stronger than in the current warm and more productive regime [16, 21, 36]. Therefore, foodweb mediated system responses to perturbations in key species was both expected, and observed in the model system. In summary, we conclude that the model performed well, and that the responses to the perturbations gave credibility to the model. There is of course still work to be done on model development. Parameterization on some of the components, such as killer whale should be improved, and significant drivers such as climate and fisheries must be added before both assessing model skill (e.g. the models ability to recreate observed development in species and systems) and testing management strategies in a climate change perspective.

Identifying critical variables in the model is important for the ecosystem-based management aspects of end-to-end models. Knowing where the largest uncertainty of the model output lies ([58] can identify those processes where more studies and observations are needed to improve the model. That way, the uncertainty of the model connected to that particular parameter can be reduced, or at least quantified. Here, we concluded that growth rate was the most influential parameter, and that the system had a strong bottom-up control. We also noticed that the components position in the foodweb to a large degree defined its sensitivity to the parameter perturbations that was done.

By limiting our analysis within the full model to just the key groups we simplified an exceptionally challenging task, but we feel it is justified as the key species studied here make up a substantial part of the whole ecosystem biomass that is of most interest to users and stakeholders (i.e. omitting the large biomasses of bacterial and benthic habitats). Theoretically the ‘perfect’ solution would be to do similar perturbations on all components of the system, however this was not feasible within the scope of the study. A thorough evaluation of such complex and comprehensive models must contend with large amounts of output results, while often lacking independent observations of the whole system with which to cross check the validity of the model projections [39, 59, 60]. The situation is even more challenging with a model system like Atlantis, where a more sophisticated analysis of model skills is effectively hindered by the large computational costs that it will require to run a global sensitivity study [11]. Even with modern computing infrastructure it would take 1000s of years to exhaustively run all potential parameter permutation combinations. It is common knowledge that local, qualitative sensitivity studies do not identify all weak spots in the model [61, 58]. However, we find that such approaches are still a useful first pragmatic step to identifying parameters that are a key to system sensitivity.

Sensitivity analysis of end-to-end models of this degree of complexity is not straightforward, as we have shown here, but should be a part of evaluating how the model performs [62]. By including both sensitivity and skill-assessment in the model evaluation, we build trust in the model system. These models are the only way of evaluating trade-offs in management strategies, and therefore play an important role in every ecosystem striving to move towards a more ecosystem based management.
